# Progress in the Treatment of Migraine Attacks: From Traditional Approaches to Eptinezumab

**DOI:** 10.3390/ph14090924

**Published:** 2021-09-13

**Authors:** Damiana Scuteri, Giacinto Bagetta

**Affiliations:** 1 Pharmacotechnology Documentation and Transfer Unit, Preclinical and Translational Pharmacology, Department of Pharmacy, Health and Nutritional Sciences, University of Calabria, 87036 Rende, Italy; 2 Regional Center for Serious Brain Injuries, S. Anna Institute, 88900 Crotone, Italy

**Keywords:** migraine attacks, triptans, gepants, ditans, potassium channels blockers, calcitonin gene-related peptide (CGRP), eptinezumab

## Abstract

Migraine is the second cause of disability and of lost years of healthy life worldwide. Migraine is characterized by recurrent headache attacks and accompanying disabling symptoms lasting 4–48 h. In episodic migraine, attacks occur in less than 15 days per month and in chronic migraine, in more than 15 monthly days. Whilst successful translation of pharmacological discoveries into efficacious therapeutics has been achieved in the preventative therapy of chronic migraine, treatment of acute migraine suffers the lack of effective advancements. An effective treatment affords complete freedom from pain two hours after therapy and provides the absence of the most bothersome symptom (MBS) associated with migraine after 2 h. However, available anti-migraine abortive treatments for acute attacks do not represent an effective and safe treatment for all the populations treated. In particular, the most used specific treatment is represented by triptans that offer 2-h sustained freedom from pain achieved in 18–50% of patients but they are contraindicated in coronary artery disease, stroke and peripheral vascular disease due to the vasoconstriction at the basis of their pharmacologic action. The most novel therapies, i.e., gepants and ditans, are without sufficient post-marketing data for secure use. Here, an attempt is proposed to analyse the rational basis and evidence in favour of investigating the efficacy and safety in acute migraine attacks of eptinezumab, i.e., monoclonal antibody (mAb) directed towards calcitonin gene-related peptide (CGRP) unique for intravenous infusion administration.

## 1. Introduction

**Figure 1:**
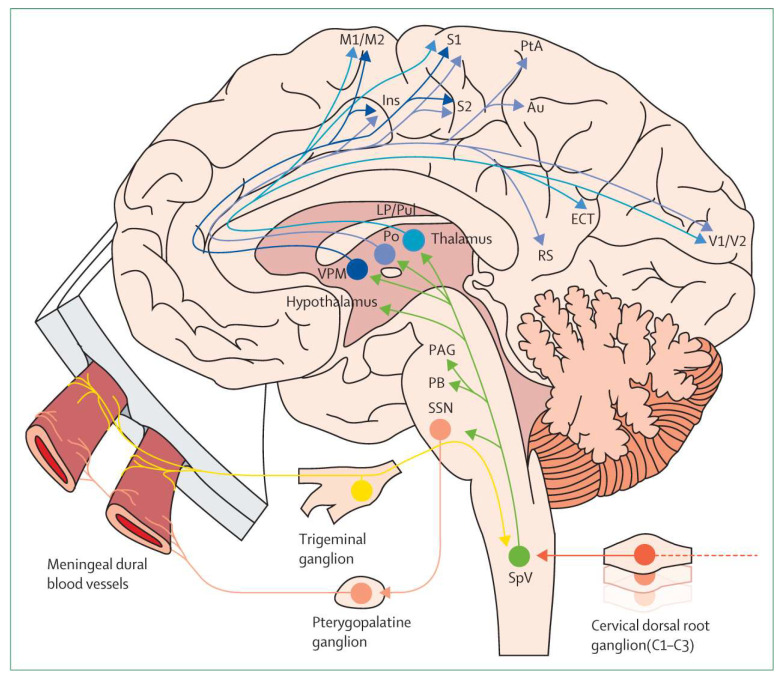
The complex trigeminovascular system and the activation of migraine. Sensory nociceptors innervating dural meningeal arteries undergo activation due to chemical or physical stimuli and project to second- and third-order neurons inducing migraine and sensitization. Au = Auditory cortex. ECT= ectorhinal cortex. Ins = insular cortex. LP = lateral posterior thalamic nucleus. M1 = primary motor cortex. M2 = secondary motor cortex. PAG = periaqueductal gray. PB = parabrachial nucleus. Po = posterior. PtA = parietal association cortex. Pul = pulvinar. RS = retrosplenial cortex. S1 = primary somatosensory cortex. S2 = secondary somatosensory cortex. SpV = spinal trigeminal nucleus. SSN = superior salivatory nucleus. V1 = primary visual cortex. V2 = secondary visual cortex. VPM = ventral posteromedial (adapted with permission from [6]).

Some 716.8 million people aged 15–49 years are affected by migraine which is the second cause of disability and of lost years of healthy life worldwide and the first within the female population [1]. The International Classification of Headache Disorders 3rd edition (ICHD-3) defines migraine as a primary headache disorder characterized by episodes which can occur with (in 1/3 of cases) or without aura, usually preceded of hours or days (premonitory phase) or associated with transient focal neurological symptoms (visual, sensory, language and brainstem disturbances) [2]. Some of these symptoms due to altered balance between sympathetic and parasympathetic systems can last over the attack to the postdromal phase [3]. Common clinical features are its unilaterality, pulsatility and moderate-to-severe intensity of throbbing pain; it is accompanied by nausea, photophobia and/or phonophobia [2]. Individual chronotypes at the basis of circadian timing of migraine attacks based on chronobiological mechanisms have been suggested [4]. The fundamental relay involved in migraine pathophysiology is represented by thalamic nuclei as the core of the trigeminovascular system [5]. Nociceptive fibres from the trigeminal ganglion, especially in the ophthalmic branch of the trigeminal nerve, innervate the dura mater and the cerebral arteries. Through the release of vasoactive neuropeptides (e.g., calcitonin gene-related peptide, (CGRP), and pituitary adenylate cyclase-activating polypeptide, (PACAP)), ATP, glutamate and NO, the latter fibres convey painful chemical or physical stimuli from pericranial skin and muscles to second-order neurons within the trigeminal cervical complex, from which projections to the brainstem, thalamic nuclei and hypothalamus and successively from these to cortical regions originate the attack [3]. The trigeminovascular pain pathway is illustrated in Figure 1.

## 2. Therapies for Migraine Attacks and Unmet Medical Needs

According to the International Headache Society (IHS) recommendations for the clinical trials, testing pharmacological agents for the acute treatment of migraine, the testing agents needs to provide 2 h after therapy complete freedom from pain and absence of the most bothersome symptom (MBS) associated to migraine [7]. Therefore, the ideal treatment of migraine attacks should provide the highest speed of action onset against pain and the MBS, which should be long-lasting to prevent relapse within 24 or 48 h from the initial treatment (sustained pain freedom), together with a good safety profile allowing for adherence. Currently, the horizon of evidence-based analgesics for the acute treatment of migraine has expanded but the complete and safe relief from acute attacks in a high percentage of patients is still an unmet medical need. Novel acute treatments are added to the established therapeutic armamentarium that can be distinguished in non-specific, symptomatic treatments and specific anti-migraine drugs for the abortion of acute attacks.

### 2.1. Not Specific Symptomatic Treatments

Acetaminophen and non-steroidal anti-inflammatory drugs (NSAIDs), in particular aspirin, diclofenac, and ibuprofen, represent the first-line treatment for mild-to-moderate acute episodes. All the NSAIDs have met the primary endpoint of the disappearance of pain after 2 h, but in a small percentage of patients compared to placebo [8,9]. A Cochrane systematic review and its update have demonstrated that ibuprofen confers relief in half of the population, but the complete disappearance of headache pain and symptoms only in a minority of migraineurs [10,11]. Additionally, diclofenac provides pain-free time only in a minority of patients [12]. Aspirin has resulted effective for acute migraine sufferers, but for the reduction of the symptoms nausea and vomiting the addition of metoclopramide 10 mg is useful [13,14]. Naproxen has proven effective in only 2 out of 10 migraine sufferers, not being a stand-alone clinically useful treatment [15]. Another important issue of NSAIDs is represented by the adverse reactions, including gastrointestinal and cardiovascular serious side effects also induced by cyclooxygenase 2 (COX-2) inhibitors. In fact, the PRECISION-ABPM (Prospective Randomized Evaluation of Celecoxib Integrated Safety vs. Ibuprofen or Naproxen Ambulatory Blood Pressure Measurement) Trial has demonstrated hypertension in 23.2% patients for ibuprofen, 19.0% for naproxen and 10.3% for celecoxib with physiological baseline blood pressure values [16]. Among the most ancient therapies for migraine, the ergot derivatives assume a historical significance, leading to the development of dihydroergotamine now available as injectable (intravenous/intramuscular/subcutaneous) and intranasal formulations [17]. The use of the latter drug is not feasible without an anti-emetic and in patients with cardiovascular disease and hypertension. In fact, they are characterized by an increased rate of catheter-associated venous thromboses [18] and a case of likely vasospastic angina in the absence of coronary artery disease has been described [19]. In course of severe migraine attacks, also opiates can be used, paying attention to the mortality rate for abuse of these drugs often used in combination with gabapentinoids [20].

### 2.2. Anti-Migraine Abortive Treatments for Acute Attacks

Triptans are selective 5-HT_1B/1D/(1F)_ agonists used as a gold standard treatment for moderate-to-severe acute migraine attacks. Unfortunately, some 40% of migraine sufferers do not respond to the latter treatment and percentages of complete pain relief after 2 h are variable, as it is the tolerability profile within the class [21,22]. In fact, the first triptan to be used since the early 1990′s, sumatriptan, is characterized by poor oral bioavailability, ~14% (~100% after subcutaneous administration) and lipophilicity [23], thus leading to zolmitriptan, eletriptan, frovatriptan, rizatriptan and almotriptan, the second generation with improved pharmacokinetics (PK) [24]. Among these, almotriptan is one of the newest triptan with a favourable PK profile, 70% oral bioavailability, not affected by gender or food [25], and not bearing important drug-to-drug metabolic interactions apart from with CYP3A4 inhibitors [24]. These characteristics may in part explain its frequency of use in real-world settings [26]. Intranasal zolmitriptan presents fast cerebral penetration and PK advantages over the other routes of administration [27]. In fact, a nasal spray is available for sumatriptan and for zolmitriptan in Italy [28]. A network meta-analysis has compared the effectiveness of the various triptans and of not specific symptomatic treatments [29]. Based on this study, triptans afforded 2-h sustained freedom from pain in 18–50% of patients [29]. Data of pain relief and freedom at 2 h for each triptan and for the other symptomatic treatments [29] are reported in Table 1.

Apart from the 40% of triptan users that reported unmet needs [30], it is very important to underline that triptans exert their therapeutic action through the vasoconstriction of cranial vessels (via agonism at 5-HT_1B_ receptor and an indirect effect on CGRP), consequently inhibiting transmission throughout the trigeminocervical system. This characteristic makes them contraindicated with coronary artery disease, stroke, and peripheral vascular diseases [31]. Therefore, the research has moved towards drugs that do not induce vasoconstriction, i.e., gepants that are CGRP receptor antagonists and ditans that are 5-HT_1F_ receptor agonists. Since these drugs have been recently approved, longer clinical post-marketing experience is needed for the accurate determination of adverse events, mainly for people suffering from cardiovascular diseases and during pregnancy [32]. Ubrogepant has been investigated in the NCT03461757 double-blind, randomised, placebo-controlled, multicentre phase III trial [33]; in the NCT03237845 multicentre, double-blind, phase III clinical trial [34]; in the “Efficacy, Safety, and Tolerability Study of Oral Ubrogepant in the Acute Treatment of Migraine” (ACHIEVE I) (NCT02828020) phase III, multicentre, randomized, double-blind, placebo-controlled single attack study [35] and in the phase III, multicentre, randomized, double-blind, placebo-controlled, single-attack, (ACHIEVE II) (NCT02867709) [36], it was discovered that pain freedom occured at 2 h in 19.2–21.8% of patients, based on the dose, and freedom from the migraine-associated MBS at 2 h in 34.1–38.9% of patients. Data for rimegepant efficacy obtained from the double-blind, randomised, placebo-controlled, multicentre phase III trial NCT03461757 [33] and from the multicentre, double-blind, phase III clinical trial NCT03237845 [34] demonstrated that some 19.6–21% patients achieved pain freedom at 2 h and some 35.0–37.6% obtained freedom from the migraine-associated MBS. Lasmiditan is the first ditan to be used and it has demonstrated efficacy in terms of providing pain freedom at 2 h in 10.0–32.2% of patients with 11.1–40.9% patients achieving freedom from migraine-associated MBS at 2 h [37,38]. In fact, it has provided significant efficacy, meeting key primary and secondary efficacy endpoints at 2 h in two randomized, double-blind, placebo-controlled, phase III clinical trials, that are “A Study of Two Doses of LAsMiditan (100 mg and 200 mg) Compared to Placebo in the AcUte Treatment of MigRAIne” (SAMURAI) (NCT02439320) and “A Study of Three Doses of Lasmiditan (50 mg, 100 mg and 200 mg) Compared to Placebo in the Acute TReaTment of MigrAiNe” (SPARTAN) (NCT02605174) in adults without aging limitations [37,38]. The following phase III, randomized, open-label, multi-attack study, “An Open-label, LonG-term, Safety Study of LAsmiDItan (100 mg and 200 mg) in the Acute Treatment Of MigRaine” (GLADIATOR) (NCT02565186) [39], has investigated the incidence of treatment-emergent adverse events (TEAEs) in 132 elderly subjects enrolled to the treatment (54 to placebo) from SAMURAI and SPARTAN trials and also had 85 elderly subjects from GLADIATOR itself since this population is often excluded from migraine studies. Comparability of TEAEs incidence in elderly and non-elderly has been observed in this limited sample, with at least one TEAE occurring in 36% and 35% in pooled SAMURAI + SPARTAN and in 49% and 38% in GLADIATOR of non-elderly and elderly, respectively [39]. Therefore, future studies are needed to investigate the durability of the effectiveness and the long-term safety of gepants and ditans in the different patient populations, particularly those suffering from cardiovascular diseases and in pregnancy, in comparison with other drugs.

## 3. Novel Perspectives and Future Directions: Focus on Eptinezumab

**Figure 2:**
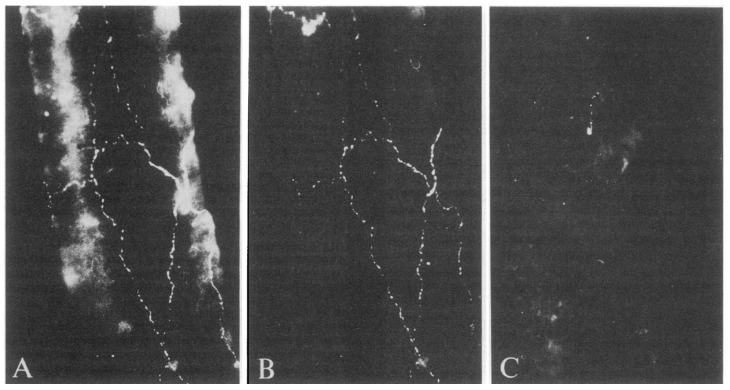
The immunoreactivity of calcitonin gene-related peptide (CGRP) around cerebral blood vessels. (**A**) Network of CGRP immunofluorescence in fibres innervating the cerebral arterioles. (**B**) Colocalization with substance P (SP). (**C**) Almost complete absence of CGRP immunofluorescence in cerebral arterioles after ipsilateral trigeminal nerve surgical lesion 14 days before sacrifice (adapted with permission from [45]).

The therapeutic options described above demonstrate, with a large degree of strength, the evidence of the efficacy (limited only for opioids [40]) and the great translational success of the research effort of the last 30 years; however, a safe abortive treatment for acute attacks effective in most patients is still lacking. Recently, potassium channels have gained interest in the field of migraine. Among migraine attack triggering molecules, e.g., CGRP, drugs that open large-conductance calcium-activated potassium (BK_Ca_) and ATP- sensitive potassium (K_ATP_) channels [41,42] on vascular smooth muscles cells of intracranial arteries have been recently identified as potential inducers of potassium efflux with following vasodilation and signalling of trigeminal nociceptors [43]. While further studies explore the role of the blockers of these channels, the story of monoclonal antibodies (mAbs) directed towards CGRP (eptinezumab, fremanezumab, and galcanezumab) and its canonical receptor (erenumab) has not come to an end and therapeutic innovation can be anticipated. In fact, mAbs have revolutionized the therapy of several neurologic diseases, acting through direct mechanisms in the case of migraine [44]. The presence of CGRP immunoreactive fibres around cerebral vessels originating from the trigeminal ganglion has been demonstrated (Figure 2) shedding light on the neurogenic activities exerted by the trigemino-cerebrovascular system [45].

However, the site of action of anti-CGRP mAbs, peripheral or intracerebral, is still a debated issue though there is pharmacological evidence in support of a central action [46]; the latter strengthens the need for deepened investigation to understand the therapeutic potential of the latter biologics. Erenumab efficacy has been studied in the randomized, double-blind, placebo-controlled, phase III “Study to Evaluate the Efficacy and Safety of Erenumab (AMG 334) Compared to Placebo in Migraine Prevention (ARISE)” (NCT02483585) [47], showing significant differences with the placebo in monthly migraine days (MMDs) and a ≥50% reduction in 39.7%, with the typical side effects of these treatments most frequently consisting in upper respiratory tract infections and injection site pain. The phase III, randomized, double-blind, placebo-controlled “Study to Evaluate the Efficacy and Safety of Erenumab (AMG 334) in Migraine Prevention” (STRIVE) (NCT02456740) [48] has highlighted a 3.2–3.7 dose-related MMDs reduction. The efficacy of erenumab in episodic migraine non-responding to previous treatments has been assessed in 12 week-phase IIIb “Study Evaluating the Effectiveness of AMG 334 Injection in Preventing Migraines in Adults Having Failed Other Therapies” (LIBERTY) (NCT03096834) [49] reporting its efficacy in these difficult-to-treat patients. The effectiveness in episodic (HALO EM, NCT02629861) and chronic (HALO CM) migraine has been observed also for fremanezumab, even in the reduction of acute medications [50,51,52]. Additionally, efficacy in patients whose treatments had previously failed has been documented for fremanezumab in the phase IIIb multicentre, randomized, double-blind, parallel-group, placebo-controlled (with an open-label period) “Efficacy and Safety Study of Fremanezumab in Adults With Migraine” (FOCUS) (NCT03308968) study [53]. Moreover, the 52-week, multicentre, randomized, double-blind, parallel-group study (NCT02638103) has confirmed the long-term safety and tolerability of fremanezumab [54]. Additionally, galcanezumab has provided evidence for efficacy in the prevention of episodic migraine in the “Evaluation of LY2951742 in the Prevention of Episodic Migraine 1 and 2” (EVOLVE-1 and EVOLVE-2) double-blind, randomized, placebo-controlled trials (NCT02614183, NCT02614196) [55,56] and of chronic migraine in the phase III “Evaluation of Galcanezumab in the Prevention of Chronic Migraine” (REGAIN) study (NCT02614261) [57], showing long-term safety in the phase III, long-term, open-label NCT02614287 safety study [58], and has shown some reduced efficacy post-treatment [59]. The effectiveness of fremanezumab in treatment-resistant migraine has been assessed in patients aged 18–75 years in the “Study of Galcanezumab (LY2951742) in Adults With Treatment-Resistant Migraine (CONQUER)” (NCT03559257) [60]. The phase III, randomized, double-blind, placebo-controlled Prevention of Migraine via Intravenous ALD403 Safety and Efficacy 1 (PROMISE-1) (NCT02559895) clinical trial [61] has demonstrated the significant effectiveness of the intravenous administration of eptinezumab with a higher number of patients with a ≥50% or ≥75% reduction of episodic migraine compared to the placebo during one year of treatment, in which it was well tolerated. The phase III, randomized, double-blind, placebo-controlled PROMISE-2 (NCT02974153) study has demonstrated significant improvement and preventative efficacy in patients suffering from chronic migraine in 24 weeks [62]. The long-term (2 year-long) safety of eptinezumab in patients with chronic migraine has been evaluated in the study, “An Open Label Trial of ALD403 (Eptinezumab) in Chronic Migraine” (PREVAIL) (NCT02985398) [63], which has confirmed the benefit in chronic migraine associated with a favourable safety profile 24 week-immunogenicity up to non-detectable levels. The PK characteristics of anti-CGRP mAbs may account for some differences in their therapeutic application. In fact, eptinezumab is the only anti-CGRP mAb administered for intravenous infusion adding a faster action [64] due to the rapid reaching of maximum plasma concentration (Cmax; within around 30 min, i.e., the end of the infusion) [65,66], to the long half-life (characteristic of the CGRP mAbs class of biologics), allowing the dilated frequency of administration [67]. In particular, eptinezumab intravenous administration occurs every 3 months and it has demonstrated a promising preventive effect in episodic migraine [68]. The phase 3, multicentre, parallel-group, double-blind, randomized, placebo-controlled trial NCT04152083 [69] has assessed the efficacy of the infusion of eptinezumab during an active migraine attack in patients eligible for preventive migraine treatment based on ICHD-3 criteria: the co-primary endpoints were time to headache pain freedom and time to absence of MBS, both expressed as median time. The results of this study are noteworthy since eptinezumab provided headache pain freedom 4 h after the start of infusion and the absence of MBS 2 h after the start of infusion [69]. Furthermore, eptinezumab has been found to afford faster time to headache pain relief, sustained headache pain freedom and delayed time to next attack with a favourable safety profile [69]. *Status migrainosus* is a debilitating attack lasting longer than 72 h, usually treated with parenteral drugs (e.g., ketorolac or dihydroergotamine); eptinezumab could be very useful for its rapid action and its preventative efficacy. In fact, anti-CGRP mAbs show a better benefit/risk ratio in comparison with established therapies for episodic and chronic migraine [70]. Therefore, although not approved for acute treatment, eptinezumab is endowed with the potential to abort migraine attacks: these data, in conjunction with the PK characteristics, do support the need for a future clinical trial designed to investigate the efficacy and safety of the latter mAb in the treatment of acute attacks occurring 2–8 times per month with at least 48 h of pain freedom between attacks and fewer than 15 headache days per month, according to current criteria for acute attacks clinical trials [7].

## 4. Conclusions

The search for an effective and safe abortive treatment for migraine attacks affording efficacy to most sufferers still represents a challenge. In fact, triptans provide 2-h of sustained freedom from pain, this was observed in 18–50% patients [29] and the most novel options, i.e., gepants and ditans, need long-term post-marketing reporting of data for adverse events in people affected by cardiovascular diseases and in pregnant women [32]. Pharmacologic [64] and clinical data (see NCT04152083 clinical trial) [69] form the rational basis for the study of the latter anti-CGRP mAb, being administered for intravenous infusion and reaching Cmax at the end of the infusion, in acute attacks. Therefore, a clinical trial with the adequate design to investigate the efficacy and safety of eptinezumab in the treatment of acute attacks occurring 2–8 times per month with at least 48 h of pain freedom between attacks and fewer than 15 headache days per month [7] is needed. Moreover, due to the epidemiology of migraine, generally the eldest population are not included in clinical trials for current abortive treatments, e.g., triptans [26]: this issue is worsened in patients affected by dementia who do not receive adequate treatment for chronic pain [71,72,73]. Hence, this clinical trial should include this fragile population. Another pivotal problem is represented by patients suffering from mild traumatic brain injury who can develop post-traumatic headache in which the pathogenesis involves CGRP as well [74]. The investigation of eptinezumab could represent a completely new pattern of treatment for acute attacks, including effective prevention of following episodes.

## Figures and Tables

**Table 1:** Percentage of Patients, Odds ratio (OR) and 95% Credible Interval (CrI) with headache relief or freedom from pain at 2 h with a standard dose of triptans and not specific symptomatic treatments administered for oral route.

Drug (Standard Dose)	2-h Headache Relief	2-h Freedom from Pain
Almotriptan (12.5 mg)	48.30%, 2.56 (2.0–3.3)	24.50%, 2.73 (2.1–3.6)
Eletriptan (40 mg)	60.40%, 4.19 (3.5–5.0)	39.20%, 5.43 (4.3–6.9)
Frovatriptan (2.5 mg)	42.40%, 2.02 (1.5–2.7)	34.70%, 4.48 (2.8–7.3)
Naratriptan (2.5 mg)	44.50%, 2.20 (1.5–3.2)	17.50%, 1.79 (1.1–2.8)
Rizatriptan (10 mg)	57.10%, 3.66 (3.0–4.5)	36.60%, 4.86 (3.9–6.2)
Sumatriptan (50 mg)	49.70%, 2.71 (2.4–3.1)	27.70%, 3.22 (2.7–3.8)
Zolmitriptan (2.5 mg)	50.00%, 2.75 (2.3–3.3)	27.10%, 3.14 (2.5–4.0)
Acetaminophen (1000 mg)	51.70%, 2.94 (1.2–7.2)	22.20%, 2.41 (0.9–6.6)
Aspirin (1000 mg)	46.10%, 2.35 (1.3–4.2)	24.00%, 2.66 (1.3–5.3)
Ergot derivatives (2 mg)	38.40%, 1.71 (1.2–2.5)	15.50%, 1.55 (0.8–3.2)

## Data Availability

No new data were created or analysed in this study. Data sharing is not applicable to this article.
